# The effects of temperature and donor piglet age on the transcriptomic profile and energy metabolism of myoblasts

**DOI:** 10.3389/fphys.2022.979283

**Published:** 2022-09-21

**Authors:** Katharina Metzger, Claudia Kalbe, Puntita Siengdee, Siriluck Ponsuksili

**Affiliations:** ^1^ Research Institute for Farm Animal Biology (FBN), Institute of Muscle Biology and Growth, Dummerstorf, Germany; ^2^ Research Institute for Farm Animal Biology (FBN), Institute of Genome Biology, Dummerstorf, Germany

**Keywords:** satellite cells, myoblasts, temperature, pig, transcriptome, energy metabolism

## Abstract

Rapid climate change is associated with frequent extreme heat events and the resulting thermal stress has consequences for the health, welfare, and growth of farm animals. The aim of this study was to characterize the transcriptional changes and the effects on energy metabolism in proliferating porcine myoblasts derived from piglets of different ages, representing differences in thermoregulatory abilities, and cultivated below (35°C) and above (39°C, 41°C) the standard cultivation temperature (37°C). Satellite cells originating from *Musculus rhomboideus* of piglets isolated on days 5 (P5, thermolabile) and 20 (P20, thermostable) of age were used. Our expression analyses highlighted differentially expressed genes in porcine myoblasts cultures under heat or cold induced stress. These gene sets showed enrichment for biological processes and pathways related to organelle fission, cell cycle, chromosome organization, and DNA replication. Culture at 35°C resulted in increased metabolic flux as well as a greater abundance of transcripts of the cold shock protein-encoding gene *RBM3* and those of genes related to biological processes and signaling pathways, especially those involving the immune system (cytokine–cytokine receptor interaction, TNF and IL-17 signaling pathways). For cultivation at 39°C, differences in the expression of genes related to DNA replication and cell growth were identified. The highest glutathione index ratio was also found under 39°C. Meanwhile, cultivation at 41°C induced a heat stress response, including the upregulation of *HSP70* expression and the downregulation of many biological processes and signaling pathways related to proliferative ability. Our analysis also identified differentially expressed genes between cells of donors with a not yet (P5) and already fully developed (P20) capacity for thermoregulation at different cultivation temperatures. When comparing P5 and P20, most of the changes in gene expression were detected at 37°C. At this optimal temperature, muscle cells can develop to their full capacity. Therefore, the most diverse molecular signaling pathways, including PI3K-Akt signaling, Wnt signaling, and EGFR tyrosine kinase inhibitor, were found and are more pronounced in muscle cells from 20-day-old piglets. These results contribute to a better understanding of the mechanisms underlying the adaptation of skeletal muscle cells to temperature stress in terms of their thermoregulatory ability.

## Introduction

Climate change exerts multidimensional effects on food and agricultural systems, thereby strongly influencing crop and livestock productivity (Schmidhuber and Tubiello, 2007). Thermal stress may occur under warm and cold environments. This type of stress has led to corresponding hazards due to the increasing number of extreme heat events worldwide, which in turn pose an increased risk to the growth, health, and welfare of animals in farming systems (St-Pierre et al., 2003; Baumgard and Rhoads 2013; Horton et al., 2016). Newborn piglets cannot maintain their body temperature in the first week of life (Curtis and Rogler 1970) due to lack of brown adipose tissue (Trayhurn et al., 1989; Herpin et al., 2002). Changes of the climatic and nutritional environment play an important role during this period (Schmidt and Herpin 1998). Several *in vivo* studies have investigated the effects of heat stress on the physiology (Le Bellego et al., 2002; Patience et al., 2005; Hao et al., 2014), proteomic profile (Cruzen et al., 2015) and epigenomic profile (Hao et al., 2016) in pigs.

The microenvironment of the myofibres (stem cell niche) largely directs satellite cell functions. The natural environment of the muscle fiber type and its origin play an important role in controlling satellite cell properties (Zhu et al., 2013). Additionally, donor age and species differences can affect the myogenic capacity of satellite cells *in vitro* (Gonzalez et al., 2020), while the environment can modulate satellite cell sensitivity to thermal stress. Heat stress can affect the differentiation, proliferation, muscle fiber type, protein turnover, and abundance of heat shock proteins in muscle satellite cells of pigs and chickens as well as in C2C12 myoblasts, an immortalized mouse myoblast cell line (Yamaguchi et al., 2010; Kamanga-Sollo et al., 2011). Muscle metabolism and contractile function are also sensitive to changes in temperature (James 2013). Low temperatures can lead to an energy deficit in skeletal muscle cells, resulting in an increase in mitochondrial biogenesis and ATP production (Jäger et al., 2007; Lira et al., 2010). Relatively few studies have utilized primary muscle cell cultures derived from satellite cells from farm animals to investigate the effects of temperature. Kamanga-Sollo et al. (2011) and Gao et al. (2015) investigated the effects of heat stress by a single high temperature stimulus in porcine satellite cell cultures. Meanwhile, Reed and colleagues investigated the effect of thermal stress (33°C or 43°C vs. 38°C) on the transcriptome of turkey muscle satellite cells at the proliferation (Reed et al., 2017a) and differentiating (Reed et al., 2017b) stages.

We hypothesize that satellite cell-derived cell cultures are able to mimic muscular adaptation to temperature stress as well as exhibit distinct gene expression patterns that reflect their developmental commitment and influence their responsiveness to thermal stress. Therefore, we cultured proliferating myoblasts below (35°C) and above (39°C and 41°C, respectively) the standard cultivation temperature (37°C) and evaluated the effects on the transcriptome, oxidative stress and energetic metabolism, using our well-established cell pooling approach (Metzger et al., 2020). We also investigated the molecular changes occurring in cultures of porcine primary muscle cells originating from donor piglets with different capacities for thermoregulation and cultured under different temperatures.

## Materials and methods

### Cell culture

The isolation of satellite cells from the kursiv *M. rhomboideus* of 10 female five- and 20 days old piglets and the establishment and validation of two muscle cell pools (P5, *n* = 10; P20, *n* = 10) were performed as previously described (Metzger et al., 2020). For proliferation experiments cells from both pools stored in liquid nitrogen were defrosted and cultured for 72 h at 35°, 37° (control), 39° or 41°C in growth medium with one medium change after 48 h as described by Metzger et al. (2021). A total of 1 × 10^6^ cells from each pool were seeded in 100-mm gelatin-coated culture dishes (Sarsted, Nümbrecht, Germany) for microarray analysis. To explore mitochondrial and glycolytic functional changes, 2,000 cells/well and 20 wells per pool per replicate (Seahorse XFp plate, OLS, Bremen, Germany) were used. To estimate the ratio of reduced glutathione (GSH) to oxidized glutathione (GSSG), 3,000 cells/well and 10 wells per pool per replicate were used (96 well-microplates, Sarstedt). Three replicates were generated for each experiment.

### RNA isolation, microarray experiment and analyses

Total RNA was isolated from cells after 72 h of proliferative growth using TRIzol reagent (Sigma-Aldrich) and a RNeasy Mini Kit (Qiagen, Hilden, Germany) according to the manufacturer’s instructions. Porcine Snowball Microarrays (Affymetrix, Thermo Fisher Scientific, Schwerte, Germany) containing 47,880 probe-sets were used in this study. For cDNA synthesis, 500 ng of total RNA was used and subsequent biotin labelling was performed with the Affymetrix WT plus Expression Kit (Affymetrix) and Genechip WT terminal labeling and hybridization Kit (Affymetrix) according to the manufacturer’s instructions. A total of 24 label cRNA samples (*n* = 12 per pool) were hybridized on the microarrays. Afterwards washing and scanning was performed according to manufacturer’s recommendations. Quality control was performed using Affymetrix GCOC 1.1.1. software. Expression Console software was used for robust multichip average (RMA) normalization and the detection above background (DABG) algorithm was used to detect the genes that were present. Probe sets with low signal and those that were present in less than 80% of the samples within each temperature group were excluded. After filtering, 13,226 probe sets were finally used for further analyses.

Differential expression analysis was performed using mixed model analysis in JMP genomics (version 9, SAS Institute Inc., Cary, NC, United States). Temperature (35°, 37°, 39° or 41°C), pool (P5 or P 20) and the interaction of pool and temperature were used as fixed factors. Differences between least square means (LSMs) were analyzed using Tukey-Kramer tests Adjustments for multiple comparisons were performed using the Benjamini and Hochberg (1995), and a corrected *p*-value threshold of 0.05 was set as the false discovery rate (FDR).

### Functional annotation of differentially expressed genes

To identify relevant functional categories across temperatures, pools, and pools under specific temperatures, gene ontology (GO) and KEGG pathway enrichment analysis of differentially expressed genes (DEGs) was performed using WebGestalt 2019 [WEB-based Gene SeT AnaLysis Toolkit (Liao et al., 2019)] and DAVID (v. 6.8). For DAVID, right-sided hypergeometric tests were used to calculate the *p*-values, while dot-plots generated using the R package ggplot2 were used to visualize the DAVID enrichment analysis results. *p* ≤ 0.05 was considered significant for biological processes and KEGG pathways.

### Validation of microarray results

Quantitative real-time PCR (qPCR) was used for the evaluation of the microarray results. RNA isolation, reserve transcription, and qPCR were performed as described by Kalbe et al. (2008, 2018) with following primers: amphiregulin (*AREG*, Kalbe et al., 2018), myosin heavy chain 3 (*MYH3*, Da Costa et al., 2002), TATA-box binding protein (*TBP*, Erkens et al., 2006), actin beta (*ACTB*, F - 5′ CTG​GCA​CCA​CAC​CTT​CTA​C - GGG​TCA​TCT​TCT​CAC​GGT​TG 3′), proliferating cell nuclear antigen (*PCNA*, Metzger et al., 2021), hypoxanthine phosphoribosyltransferase 1 (*HPRT1*, Erkens et al., 2006), heat shock protein 70 (*HSP70*, Kamanga-Sollo et al., 2011), insulin like growth factor binding protein 5 (*IGFBP5*, Rehfeldt et al., 2012) desmin (*DES*, Wilschut et al., 2008), follistatin (*FST*, Rehfeldt et al., 2012) 18S ribosomal RNA (*RN18S*, Lin et al., 2001) and histidine decarboxylase (*HDC*, D’Astous-Pagé et al., 2017). Normalization of qPCR data was performed with the endogenous reference gene *RN18S*, which was unaffected by the temperature (*p* = 0.121), by pool (*p* = 0.281) or by the interaction between temperature and pool (*p* = 0.656). The LSM ± standard errors (SE) of four genes *AREG, PCNA, MYH3*, and *HSP70* have been published before (Metzger et al., 2021) and was used for correlation analysis in the present study. Statistical analysis of qPCR data and Pearson’s correlation coefficient (r) analysis was performed in SAS v. 9.4 (SAS Institute Inc.).

### Bioenergetics assay and ratio of reduced/oxidized glutathione

Mitochondrial and glycolytic functions were analyzed using the Seahorse XFp Extracellular Metabolic Flux Analyzer, as described in Sajjanar et al. (2019). Mitochondrial function was assessed by the determination of the oxygen consumption rate (OCR, pmol/min/µg of protein), which included non-mitochondrial respiration, basal respiration, maximal respiration, proton leak, ATP production, and spare respiratory capacity. Whereas, the glycolytic functions of the cells were given as extracellular acidification rate (ECAR, mpH/min/µg of protein) including non-glycolytic acidification, glycolytic capacity, glycolysis and glycolytic reserve. The ratio of the reduced glutathione (GSH) and oxidized (GSSG) glutathione was determined by using the GSH/GSSG-GloTM Assay Kit (Promega, Walldorf, Germany) following the manufacturer’s instructions for adherent cells. For statistical analysis, data were subjected to analysis of variance using the MIXED procedure in SAS (version 9.4, SAS Institute Inc.). Pool (P5 or P20), temperature (35°, 37°, 39° or 41°C) and interaction of temperature and pool were used as fixed factors. Differences between the LSMs were analyzed using Tukey-Kramer tests. *p* < 0.05 were considered significant.

## Results

### The effect of cultivation temperature on the transcriptome

**FIGURE 1:**
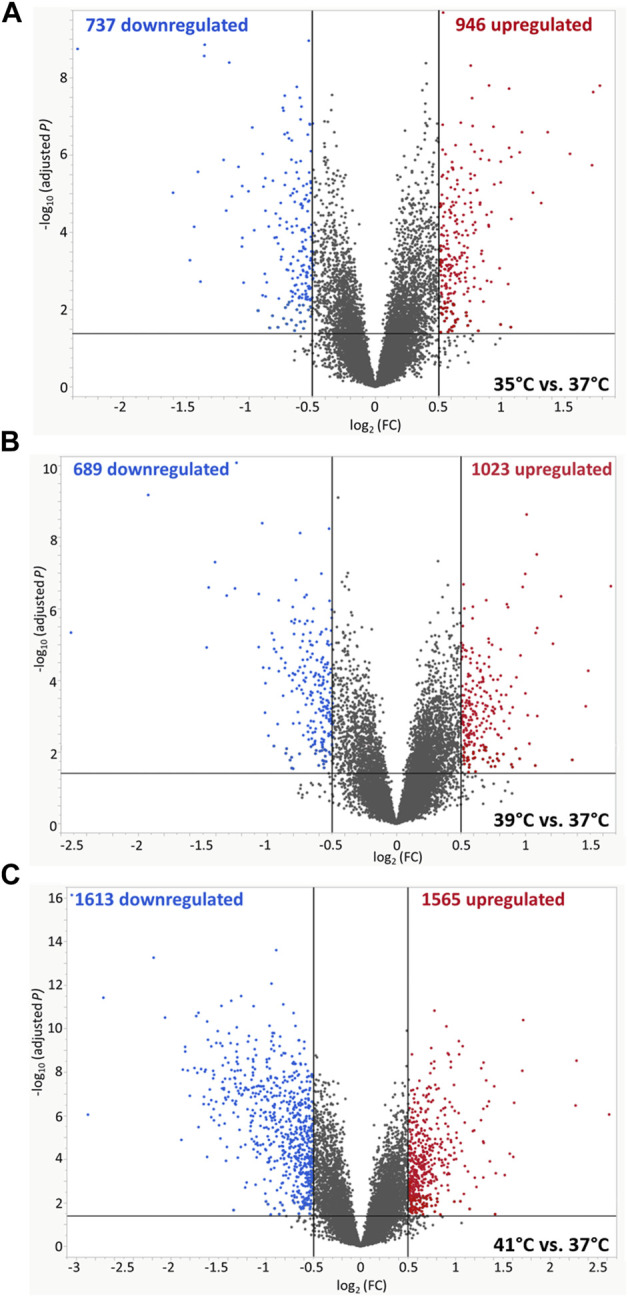
Volcano plots of differentially expressed genes (DEGs) of porcine myoblasts after 72 h of permanent cultivation at **(A)** 35°, **(B)** 39° and **(C)** 41°C compared to 37°C. The double filtering criteria are indicated by horizontal (FDR <0.05) and vertical [FC: > log_2_ (0.5) or < log_2_ (−0.5)] black lines. Blue dots represent transcripts with lower abundance (downregulated), and red dots with higher abundance (upregulated) at 35°, 39°, and 41°C compared to 37°C.

**FIGURE 2:**
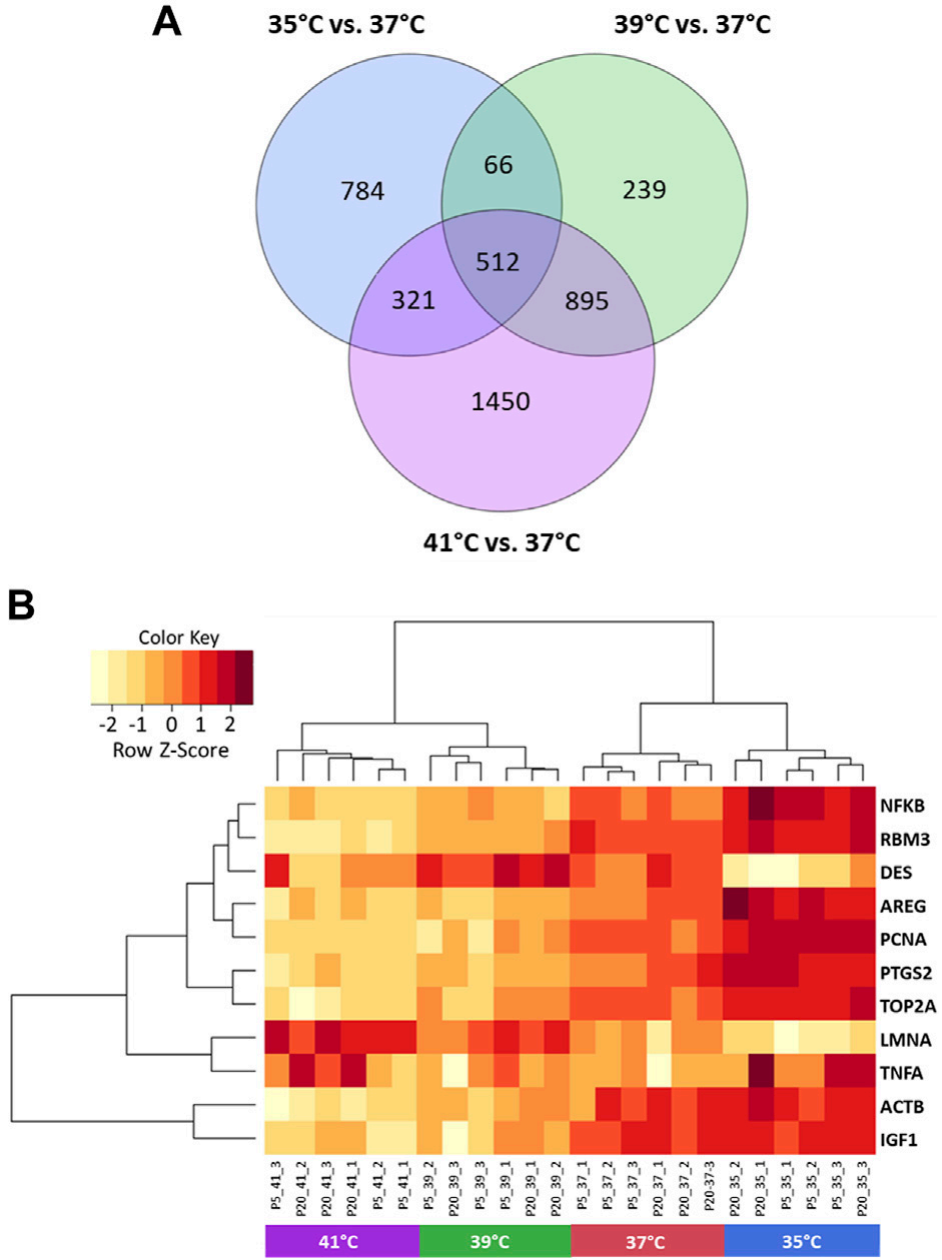
Visualization of differentially expressed genes (DEGs) of porcine myoblasts after 72 h of permanent cultivation at 35°, 39°, and 41°C compared to 37°C. Venn diagram **(A)** shows the number of DEGs for each temperature and the overlapping DEGs between different temperatures (purple 41°C vs. 37°C, green 39°C vs. 37°C and blue 35°C vs. 37°C). Heatmap **(B)** of 11 DEGs (FDR <0.05) for different permanent cultivation temperatures. The heatmap was generated using hierarchical clustering method of heatmap.2 function of ggplot2 (version 3.3.5, Wickham 2016) in the R Programming environment (version 4.0.3).

The microarray-based expression profiles of myoblasts after 72 h of proliferation at 35, 39, and 41°C were compared with those cultured at the standard cultivation temperature of 37°C (Supplementary Table S1) and the distribution of DEGs was visualized in volcano plots (Figure 1). At 35°C (Figure 1A), a total of 1,683 DEGs were found, 946 of which were upregulated and 737 downregulated. At 39 °C (Figure 1B), meanwhile, 1,712 DEGs were identified, 1,023 of which were upregulated and 689 downregulated. Most DEGs (3,178) were found when comparing myoblasts grown under 41°C with those cultured at 37°C (Figure 1C); of these, 1,565 were upregulated and 1,613 were downregulated. In addition, 512 overlapping DEGs were found among the different experimental temperature regimes (Figure 2A). For the heatmap (Figure 2B), 11 DEGs were selected that were associated with muscle structure (*DES, ACTB, LMNA*), proliferation [topoisomerase 2 alpha (*TOP2A*)*, PCNA*], immune responses [tumour necrosis factor alpha (*TNFA*)*, NFKB1*], and prostaglandin biosynthesis (prostaglandin-endoperoxide synthase 2) *PTGS2, IGF1,* and *AREG* are growth factors and RNA-binding motif protein 3 (*RBM3*) is a cold-shock marker.

**FIGURE 3:**
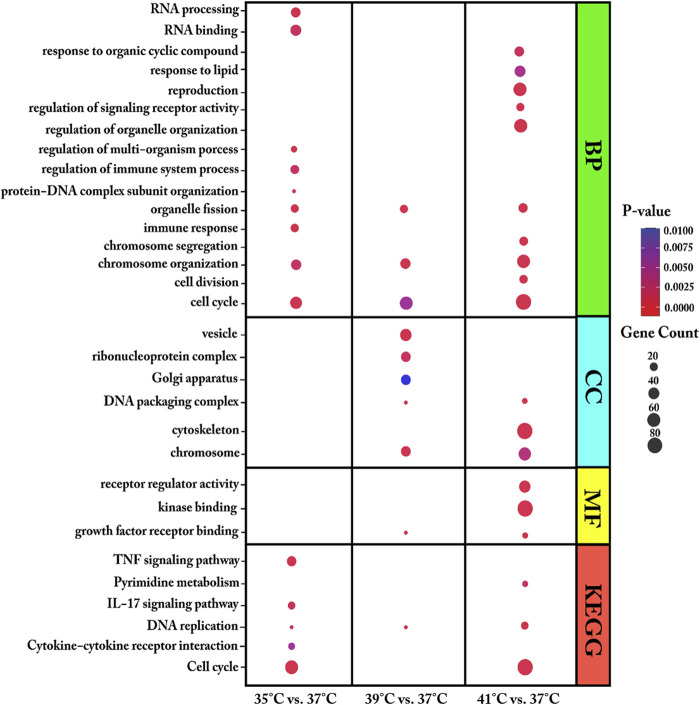
Enriched gene ontology (GO) terms of biological process (BP), molecular function (MF) and cellular component (CC) and Enriched Kyoto Encyclopaedia of Genes and Genomes (KEGG) pathways assigned to myoblasts after 72 h of proliferation permanently cultured at 35°, 39°, or 41°C compared to 37°C. The dot size embodies the number of transcripts involved in each GO term of biological process (BP), molecular function (MF), cellular component (CC) and KEGG pathway, whereas the dot’s color indicates the *p*-value.

We used DEGs from each comparison of cultured proliferating myoblasts (35, 39, or 41°C vs. 37°C) for GO and KEGG pathway enrichment analysis (Figure 3 and Supplementary Table S2). For biological process (BP), the DEGs were found to be enriched in organelle fission, cell cycle, and chromosome organization for all three comparisons. Meanwhile, at the two temperatures above the 37°C reference, the DEGs were mostly associated with the molecular function (MF) of growth factor receptor binding and the DNA packaging complex and chromosome cellular components (CC).

At 35°C, myoblasts showed an enrichment of DEGs associated with the immune response, RNA processing, regulation of immune system process, and regulation of multi-organism process. Specifically, the DEGs in myoblasts cultured at 35°C (low-temperature stress) were enriched in the KEGG pathways of cytokine–cytokine receptor interaction, interleukin 17 (IL-17) signaling pathway, cell cycle, DNA replication, signaling pathway, and TNF signaling pathway. GO enrichment analysis showed that, at 39°C, DEGs were enriched in the biological process of protein-DNA complex subunit organization and the cellular components ribonucleoprotein complex, vesicle, and Golgi apparatus. At 39°C, one KEGG pathway was identified, namely, DNA replication. The most enriched GO terms for the DEGs in myoblasts cultured at 41°C compared with those cultured at 37°C were heterogeneous and included regulation of signaling receptor activity, cell division, regulation of organelle organization, chromosome segregation, response to organic cyclic compound, reproduction, response to lipid, cytoskeleton, signaling receptor regulator activity, and kinase binding. For the highest temperature tested (41°C), three KEGG pathways were prominently represented—DNA replication, cell cycle, and pyrimidine metabolism.

### The effect of donor piglet age on the transcriptome and the interaction of donor piglet age with temperature

A total of 503 DEGs were detected between P5 and P20 after 72 h of proliferation at 35, 37, 39, or 41°C, 340 of which were upregulated and 163 downregulated (Supplementary Table S3). For the interaction (Supplementary Table S4) between P5 and P20 at 35°C, a total of 78 DEGs were found, with 40 being upregulated and 38 downregulated. A total of 198 DEGs were detected for the interaction between the pools at 37°C, 145 of which were upregulated and 53 downregulated. For the interaction between the two pools at 39°C, 51 upregulated and 87 downregulated DEGs (a total of 138) were identified. At 41°C, 119 DEGs were found, with 73 being upregulated and 46 downregulated.

**FIGURE 4:**
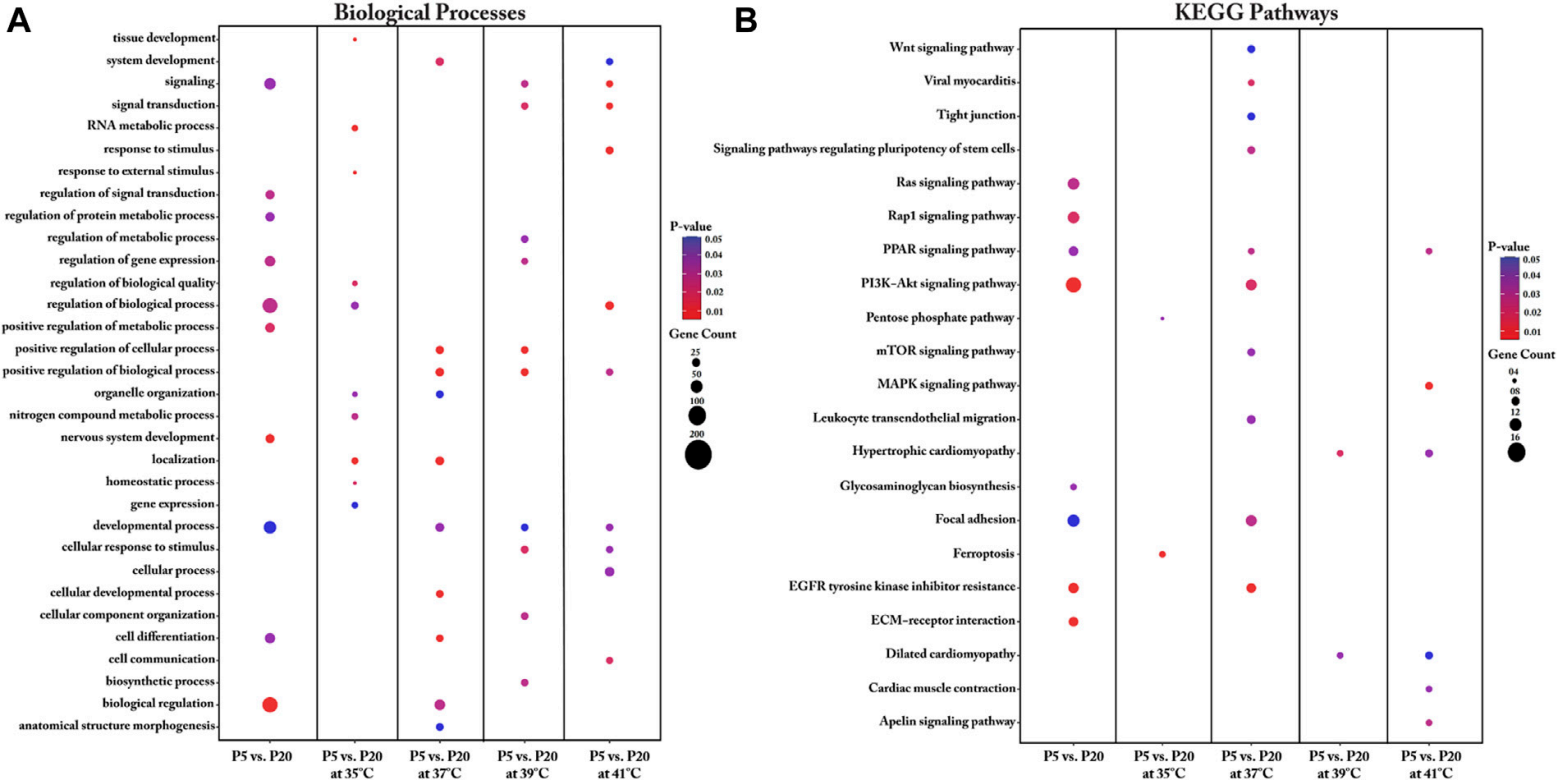
Gene Ontology **(A)** and KEGG pathway **(B)** enrichment analysis of DEGs between P5 vs. P20 at different temperatures. DEGs between P5 vs. P20 at different temperatures were subjected to DAVID (version.6.8) for functional annotation enrichment analysis. The dot size embodies the number of transcripts involved in each biological process and KEGG pathway, whereas the dot’s color indicates the *p*-value.

When comparing the transcriptomes of the donor cell pools (P5 and P20), the identified DEGs were found to be enriched in key biological processes that included regulation of signal transduction, regulation of protein metabolism, regulation of gene expression, regulation of biological processes, positive regulation of metabolic processes, nervous system development, developmental processes, cell differentiation, and biological regulation (Figure 4A, Supplementary Table S5). Our analysis further revealed several key DEGs enriched in several KEGG pathways, including the Ras signaling pathway, the Rap1 signaling pathway, the PPAR signaling pathway, the PI3K-Akt signaling pathway, glycosaminoglycan biosynthesis, focal adhesion, EGFR tyrosine kinase inhibitor resistance, and ECM-receptor interaction (Figure 4B, Supplementary Table S5).

We also undertook a functional annotation analysis of the interaction of culture temperature with the two donor cell pools. Detailed information regarding the biological processes associated with the DEGs for each interaction between pool and temperature is shown in Figure 4A and Supplementary Table S5. Most DEGs between P5 and P20 were found with cultivation under the control temperature (37°C) and were enriched in biological processes such as cellular development, cell differentiation, system development, biological regulation, anatomical structure morphogenesis, organelle organization, positive regulation of biological process, and developmental process. Interestingly, under cultivation at 39°C, the DEGs between P5 and P20 were also associated with positive regulation of biological process, positive regulation of cellular process, and developmental process. For the interaction of pools at cultivation temperatures above 37°C, the identified DEGs were enriched in the biological processes of signaling and cellular response to stimulus. For the interaction of pools at the low cultivation temperature (35°C), the genes found to be differentially expressed were enriched in biological processes related to RNA metabolic process, tissue development, and regulation of biological quality, homeostatic process, response to external stimuli, nitrogen compound metabolic process, organelle organization, and gene expression.

Important KEGG pathways affected by the donor piglet age (P5 and P20) under different cultivation temperatures were identified (Figure 4B). For the interaction between P5 and P20 under the cultivation temperature of 35°C, the DEGs were enriched in the ferroptosis and pentose phosphate pathways. Analysis of the interaction of P5 and P20 with culture at the control temperature (37°C) identified pathways associated with EGFR tyrosine kinase inhibitor resistance, viral myocarditis, PI3K-Akt signaling, focal adhesion, PPAR signaling, signaling pathways regulating the pluripotency of stem cells, mTOR signaling, leukocyte transendothelial migration, Wnt signaling, and tight junction. Most transcripts in these pathways were upregulated in P20. For the interaction between P5 and P20 under the cultivation temperature of 39°C, the DEGs were found to be associated with the hypertrophic cardiomyopathy and dilated cardiomyopathy pathways. At 41°C, meanwhile, the DEGs were enriched in the MAPK signaling pathway, the apelin signaling pathway, the PPAR signaling pathway, cardiac muscle contraction, hypertrophic cardiomyopathy, and dilated cardiomyopathy.

Eleven genes were selected for the validation of the microarray data by qPCR (Supplementary Table S6). The mRNA expression data are shown in Supplementary Table S6. The 11 genes perform a variety of functions in different molecular pathways in skeletal muscle. *MYH3, DES,* and *ACTB* are involved in muscle structure; *AREG, IGFBP5*, and *FST* are growth factors or their binding proteins; and *HSP70* is a heat shock protein. The *TBP, HPRT1*, and *PCNA* genes are associated with mitogenesis and proliferation and the *HDC* gene is associated with amino acid transport. The microarray and qPCR data showed a high correlation based on Pearson’s correlation coefficient (r), as follows: *MYH3* (*r* = 0.998, *p* < 0.001), *DES* (*r* = 0.961, *p* < 0.05), *ACTB* (*r* = 0.967, *p* < 0.05), *AREG* (*r* = 0.963, *p* < 0.05), *IGFBP5* (*r* = 0.948, *p* < 0.05), *FST* (*r* = 0.972, *p* < 0.05), *HSP70* (*r* = 0.967, *p* < 0.05), *TBP* (*r* = 0.948, *p <* 0.05), *HPRT1* (*r* = 0.991, *p* < 0.01), *PCNA* (*r* = 0.977, *p* < 0.05), and *HDC* (*r* = 0.977, *p* < 0.05).

### The effect of cultivation temperature on mitochondrial function

**FIGURE 5:**
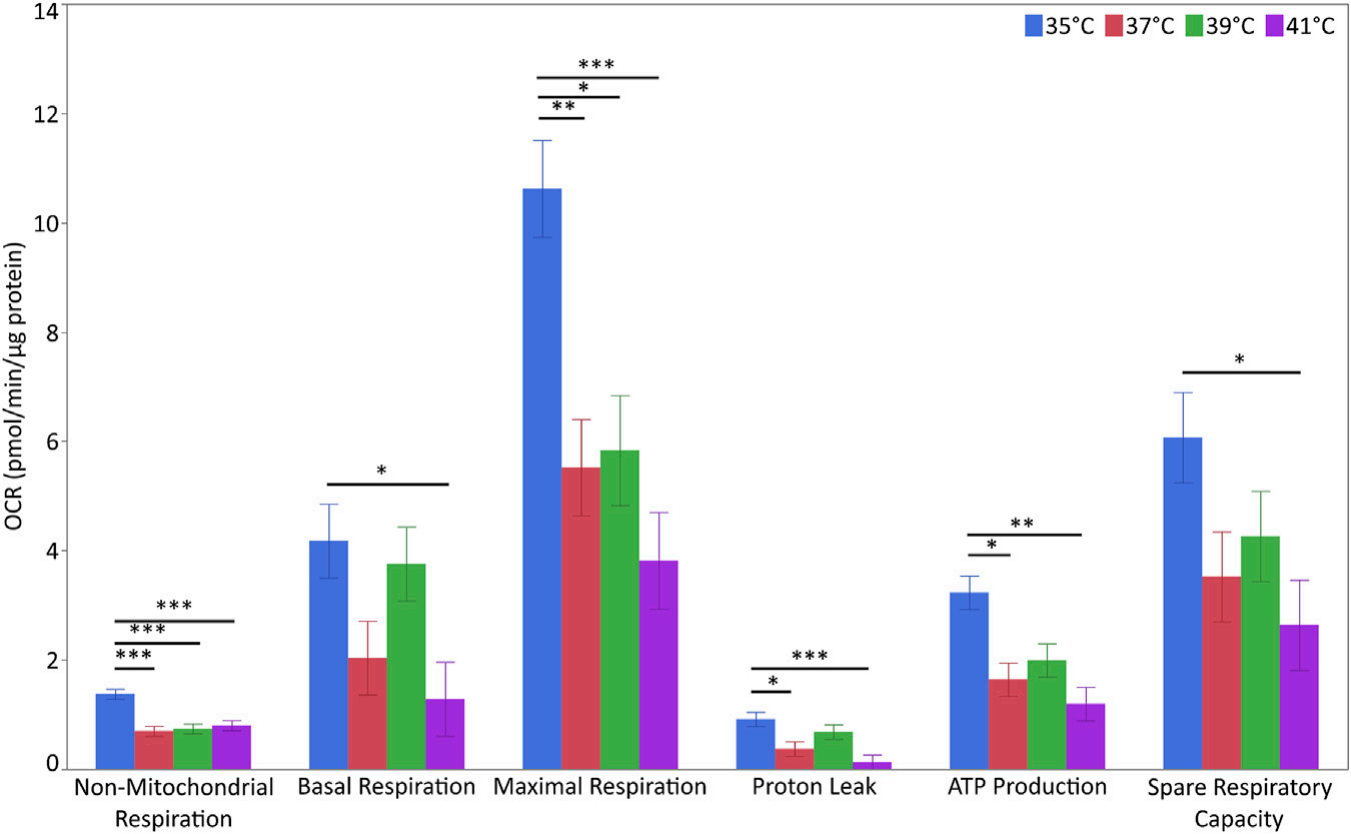
Metabolic flux in porcine myoblasts after 72 h proliferation at 35°, 37°, 39°, and 41°C. The non-mitochondrial respiration, basal respiration, maximal respiration, proton leak, ATP production and spare respiratory capacity were calculated using the Cell Mito Stress Test Kit. Data (LSM ± SE) were obtained from 10 wells per pool in each of three independent experiments. (***p < 0.001, **p < 0.01, *p < 0.05).

For the evaluation of metabolic flux, we next measured the OCR of the myoblasts (Figure 5 and Supplementary Table S7). The levels of non-mitochondrial respiration were affected by temperature (*p* < 0.001) and pool (*p* < 0.01). The highest levels were detected at 35°C (*p* < 0.001 for all comparisons). Additionally, non-mitochondrial respiration levels were higher in P5 (1.053 ± 0.065 pmol/min/µg of protein) than in P20 (0.754 ± 0.064 pmol/min/µg of protein). Basal respiration levels were affected by temperature (*p* < 0.05) but not pool. Basal respiration at 35°C was higher than that at 41°C (*p* < 0.05) but not at 37°C or 39°C. Similarly, maximal respiration levels were affected by temperature (*p* < 0.01) but not pool (*p* < 0.10). The highest respiration levels were recorded at 35°C (*p* < 0.05 vs. all the other groups). Proton leak levels were affected by temperature (*p* < 0.01) but not pool, and were higher at 35°C than at 37 and 41°C (*p* < 0.05 for both), but not at 39°C. ATP production levels displayed the same trend as the proton leak levels (*p* < 0.01). The spare respiratory capacity was unaffected by temperature or pool. However, spare respiratory capacity at 35°C was higher than that at 41°C (*p* < 0.05). None of the above parameters were affected by temperature/pool interaction.

### The effect of cultivation temperature on glycolysis and glutathione levels

**FIGURE 6:**
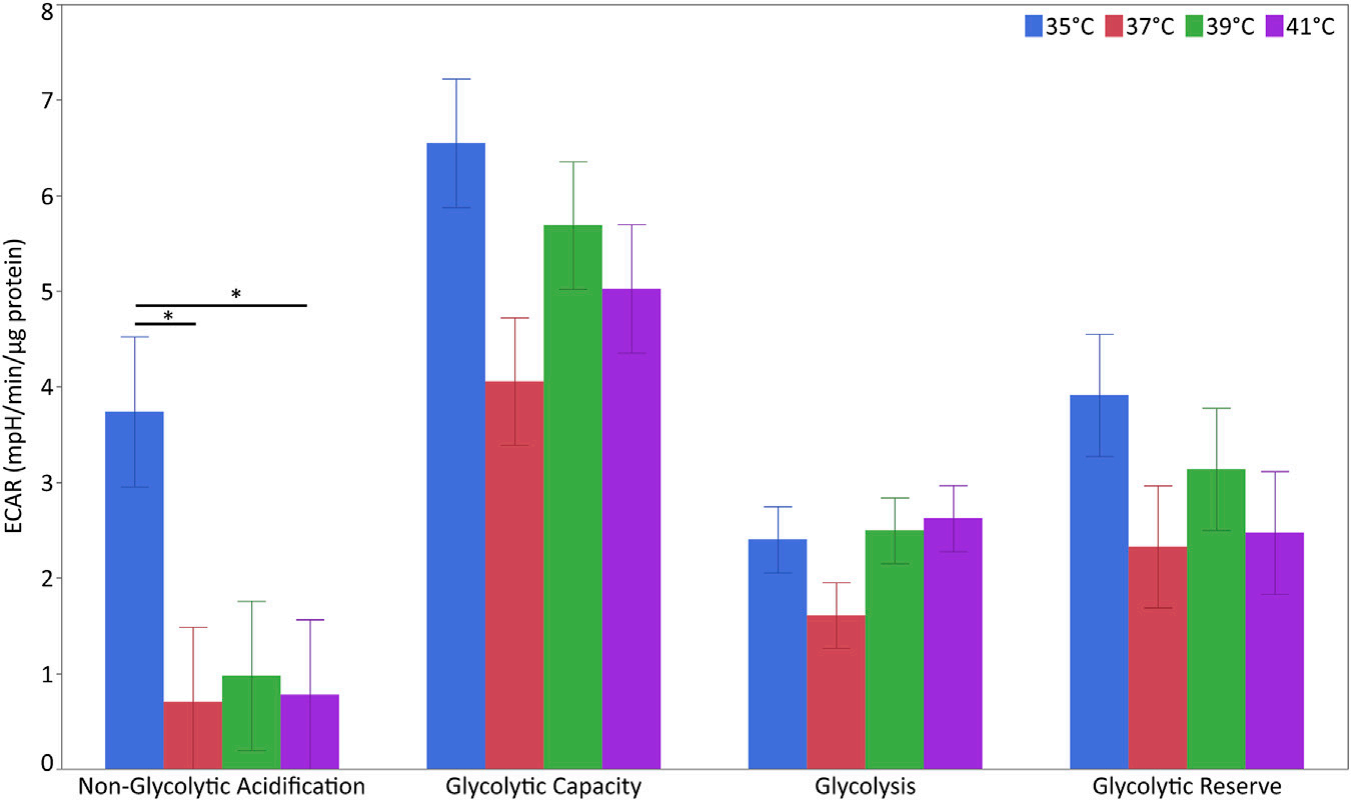
Glycolytic flux in porcine myoblasts after 72 h proliferation at 35°, 37°, 39°, and 41°C. The non-glycolytic acidification, glycolytic capacity, glycolysis, and glycolytic reserve were calculated using the Glyco Stress Test Kit. Data (LSM ± SE) were obtained from 10 wells per pool in each of three independent experiments. (*p < 0.05).

For the characterization of glycolytic stress, ECAR levels were measured at different points (Figure 6 and Supplementary Table S7). The levels of non-glycolytic acidification were affected by temperature (*p* < 0.05) but not pool or their interaction. The levels of non-glycolytic acidification were significantly higher at 35°C than at 37°C or 41°C (*p* < 0.05) but not 39°C. For glycolytic capacity, glycolysis and glycolytic reserve were unaffected by temperature, pool or their interaction.

GSH is an important scavenger of reactive oxygen species (ROS). The GSH/GSSG ratio is a valuable biomarker of oxidative stress and was found to be affected by temperature (*p* < 0.05) but not pool or temperature/pool interaction. The only difference was found between the two highest culture temperatures, with a higher ratio being detected at 39°C than at 41°C (*p* < 0.05). The results are shown in Supplementary Table S7.

## Discussion

Porcine myoblasts were cultured for 72 h at 35°, 37°, 39°, or 41°C, with 37°C being the standard cultivation temperature, and used as a reference in comparisons. In our previous study, we showed that 37°C–39°C represents the physiological range for porcine primary muscle cell culture. We have previously used cell pooling methods that allow the undertaking of long-term projects involving a wide range of experiments and numerous replications (Metzger et al., 2020), and this cell pooling method was found to reflect the average proliferative growth behavior of non-pooled cells (Metzger et al., 2020). Accordingly, for this experiment, we used cell pools derived from different animals, although this is a limitation to recognize the biological variability between the different cell donors.

A cultivation temperature of 41°C induces heat stress, whereas cultivation at 35°C results in immature myoblasts (Metzger et al., 2021). In the turkey, a cultivation of proliferating myoblasts and differentiating myotubes below and above the standard cultivation temperature leads to differences in the transcriptomic profiles of the cells (Reed et al., 2017a; Reed et al., 2017b). To the best of our knowledge, this is the first study to examine the effect of temperature stress and the interaction of thermal stress with donor age on the transcriptomic profile and mitochondrial and glycolytic cell functions of myogenic porcine cells.

### The effects of cultivation at temperatures below 37°C

Studies involving the culture of primary muscle cells below the standard cultivation temperature are rare. Most have been undertaken using primary muscle cells from birds such as the chicken or turkey (Harding et al., 2015; Clark et al., 2016; Harding et al., 2016; Clark et al., 2017). We have previously shown that porcine myoblasts can proliferate at 35°C but exhibit a different myogenic profile, characterized by higher mRNA expression levels of *PAX7, PCNA, MYF5*, and *MYOD* and higher rates of DNA synthesis, relative to myoblasts cultured at 37°C (Metzger et al., 2021). This stands in line with the enriched GO terms (cell cycle, RNA processing and RNA binding) in the present study. KEGG enrichment analysis further revealed that DNA replication- and cell cycle-related pathways were affected by cultivation at the low temperature. Additionally, we detected an increase in the levels of *RBM3*, which encodes a member of the family of cold shock proteins (Sonna et al., 2002). Ferry et al. (2011) induced a cold response in C2C12 myoblasts and also observed an increase in RBM3 protein expression. In the present study, we found that desmin (DES) levels were also reduced, and it is known that DES filament formation can be reduced by lower temperature (Chou et al., 1990). Culture of primary pig muscles at 35°C appears to induce an inflammatory response. KEGG enrichment analysis showed that the DEGs between myoblasts cultured at 35°C and those cultured at the control temperature were enriched in the TNF pathway, IL-17 pathway, and cytokine–cytokine receptor interaction, while regulation of the immune system was enriched as a GO term. TNFA, a pro-inflammatory (Nakano et al., 2006) cytokine, was also highly expressed in myoblasts cultured at 35°C. Other molecular pathways, especially signalling pathways, were also regulated at 35°C. These findings further support those of a previous study, that signaling pathways involved in cell signaling/signal transduction and cell communication/signal transduction are altered in cold-exposed satellite cells (Reed et al., 2017a).

In addition, we found an upregulation of prostaglandin-endoperoxide synthase 2 (*PTGS2*), which codes for a pro/anti-inflammatory enzyme (Funk 2001; Kadotani et al., 2009). PTGS2 is also an oxidation-associated genes and is used as a biomarker for ferrotosis (Yang et al., 2014). The upregulation of acyl-CoA synthetase long chain family member 4 (*ACSL4*) expression is also associated with sensitivity to ferroptosis (Yuan et al., 2016) and also occurs under cold temperatures, as shown in the present study. Guttridge et al. (2000) demonstrated that overexpression of TNFA activates nuclear factor-kappa B (NF-κB) in differentiating C2C12 myotubes. Similarly, in this study, we found that *TNFA* and *NFKB1* expression was upregulated in myoblasts cultured at 35°C. Further evidence that cold exposure stimulates the expression of *TNFA* in skeletal muscle was provided by Bal et al. (2017). Furthermore, TNFA/NFKB1 signalling in mitochondria was shown to be mediated *via* autoxidation at complex I or II of the respiratory chain in C2C12 myotubes (Li et al., 1999). Little and Seebacher (2016) showed that murine C2C12 myoblasts cultured at 32°C exhibit higher metabolic flux than those cultured at 37°C. This is comparable to the higher OCR values detected in our myoblasts cultured at 35°C. In addition, we previously (Metzger et al., 2021) showed that the mRNA expression of peroxisome proliferator-activated receptor gamma coactivator 1-alpha (*PPARGC1A*), a known transcriptional co-activator involved in mitochondriogenesis and mitochondrial energy metabolism (Quesnel et al., 2019), was higher in myoblasts continuously cultured at 35°C than in those cultured at 37°C.

### The effects of cultivation at temperatures above 37°C

The DEGs between porcine primary muscle cells cultured at 39°C and those cultured under the standard cultivation temperature (37°C) were primarily assigned to the GO terms of cell cycle, chromosome, DNA packaging complex, ribonucleoprotein complex, chromosome organization, protein-DNA complex subunit organization, Golgi apparatus, and vesicle.

The Golgi apparatus contributes to several cellular processes, including mitosis, DNA repair, receptor signaling and cytoskeletal regulation while Golgi-derived vesicles are key components of the intracellular communication machinery (Kalkarni-Gosavi et al., 2019). This was in line with the identified KEGG pathway of DNA replication and was also in agreement with the higher DNA content found in myoblasts cultured for 72 h at 39°C relative to those cultured at 37°C in the present study, as well as the lower *PCNA* mRNA expression levels observed in our previous study (Metzger et al., 2021). Another GO term that was enriched in porcine primary myoblasts cultivated at 39°C compared with those cultured at 37°C was growth factor receptor binding. Higher growth factor receptor expression (epidermal growth factor receptor (*EGFR*) and insulin-like growth factor 1 receptor (*IGF1R*)) with cultivation at 39°C was also found in our former study (Metzger et al., 2021). The products of both genes are known stimulators of DNA replication (Clemmons 1984; Zetterberg et al., 1984; Inoue et al., 2005; Xie et al., 2014), which is in line with the identified KEGG pathway.

After culture at 41°C for 72 h, we found that the expression of *HSPs* was increased, likely as part of a heat shock response, similar to that reported for other studies on porcine muscle cells (Kamanga-Sollo et al., 2011; Gao et al., 2015; Metzger et al., 2021). In addition, beside the higher expression of HSPs, the expression of RBM3 was downregulated (Zeng et al., 2009), which was found in the present study. Furthermore, 41°C seemed to increase the production of reactive oxygen species (ROS) but only compared to the ratio of GSSG/GHS to 39°C. Heat stress can induce mitochondrial superoxide and intracellular ROS overproduction in cultured muscle cells (Rosado Montilla et al., 2014; Kikusato et al., 2015). In addition, after continuous culture at 41°C, biological processes and down-regulated KEGG pathways including pyrimidine metabolism were enriched, which includes all enzymes involved in the synthesis, degradation, salvage, transformation, and transport of DNA, RNA, lipids, and carbohydrates (Garavito et al., 2015). Combined, these findings imply the gradual termination of myoblast proliferation. Cell cycle arrest after heat stress in porcine primary muscle cells was also found by Gao et al. (2015). In addition, we detected a marked downregulation of *TOP2A* expression in myoblasts cultured at 41°C. TOP2A is a DNA topoisomerase that is associated with RNA polymerase II holoenzyme and is a necessary component of chromatin-dependent coactivation (Mondal and Parvin 2001). Hyperthermia treatment in HeLa S3 cells (15 min at 44°C) resulted in a reduced availability of TOP2A and decreased cytotoxicity (Kampinga (1995), whereas at a later stage in DNA damage processing protection by HSPs overexpression were observed (Li 1987). These observations are in line with our previous study (Metzger et al., 2021) where we found that the expression of HSPs was increased without a concomitant change in the levels of lactate dehydrogenase (LDH), a marker of cell death, after 72 h of proliferation at 41°C. In addition, the lower expression of MYOD at 41°C observed in our previous study (Metzger et al., 2021) was indicative of prominent myoblast maturity. Previous studies also reported that when exposed to heat, myoblasts exhibit changes in the expression of genes related to muscle system development and differentiation (Reed et al., 2017a). Similarly, we identified a GO term of the cytoskeleton with the down-regulation of LMNA, a type V intermediate filament protein. Frock et al. (2005) showed that a reduction in LMNA levels resulted in decreased DES and MYOD expression in primary muscle cell cultures. As mentioned above, we also found that MYOD mRNA expression was reduced in our previous study (Metzger et al., 2021), while *DES* mRNA levels were found to be reduced in the present study. These results support the more differentiated phenotype of myoblasts at the cultivation temperature of 41°C, as evidenced by the presence of finger-like protrusions and an increase in cell size (Metzger et al., 2021).

### The effect of donor piglet age and its interaction with temperature

Thermoregulation is the ability to balance heat production and heat loss to maintain body temperature within a certain normal range, which in pigs is between 38 and 40°C, with an average of 38.8°C. Maintaining a neutral thermal environment is among of the most important physiological challenges, especially for newborn piglets. Maintaining body temperature is most difficult from 0 to 7 days of age because the piglet has no brown fat to quickly generate heat. Accordingly, we used piglets at 5 and 20 days of age, representing donors with a not yet (P5) or already fully developed (P20) capacity for thermoregulation. Understanding the biological effect of temperature stress on muscle cells in aging is important, especially in new-born piglets, which are still sensitive to environmental temperatures. Satellite cell activity was reported to be affected by the origin of donor cells, such as those obtained following maternal nutrient restriction or intrauterine growth restriction (Yates et al., 2014; Raja et al., 2016). Previous study reviewed that skeletal muscle satellite cells derived from different muscle types and different animal selected lines exert differential effects on adipogenesis when thermally challenged (Harding et al., 2015; Clark et al., 2017). Satellite cells isolated from different turkey lines display heterogeneous proliferation and differentiation abilities (Velleman et al., 2000) as well as different sensitivities to temperature changes during proliferation and differentiation (Harding et al., 2015; Reed et al., 2017a). Notably, studies investigating donor age-dependent thermoregulatory capacity remain limited.

Our study also focused on identifying differences in the transcriptomic profiles of porcine muscle cells derived from donor piglets of different ages and continuously cultured at 35, 37 (control), 39, or 41°C. Most of molecular pathways changes when comparing cells of P5 vs. P20 were found at 37°C. At this optimal temperature, the muscle cells can develop to their full capacity and show the most different molecular pathways including PPAR signaling, PI3K-Akt signaling, Wnt signaling pathways and EGFR tyrosine kinase inhibitor. Most of the transcripts enriched in these pathways were more highly expressed in P20 than in P5. However, only small changes between P5 and P20 were detected at temperatures above or below 37°C. We found that the positive regulation of the biological process, the positive regulation of the cellular process, and the developmental process were also found at 39°C, the physiological body temperature of the piglets, when comparing P5 and P20. Interesting, at 35°C, the identified DEGs were enriched in pentose phosphate pathway (PPP) as well as iron-dependent lipid peroxidation (ferroptosis), which mediates programmed cell death. The glutathione (GSH) system is the main ferroptosis-limiting pathway (Chen et al., 2021). We found significantly lower GSH level in P5 compare with P20 at 35°C (*p* < 0.001). The GSH:GSSG index, an indicator of oxidative stress, tended to be higher in P20 (*p* < 0.08). These results suggested that the muscle cells of a 5-day-old donor piglet are more susceptible to ferroptosis when exposed to cold temperatures than those of 20-day-old piglets.

## Conclusion

In this study, we focused on the transcriptional profile and energy metabolism of primary porcine muscles derived from piglets of different ages (P5 vs. P20) after continuous cultivation for 72 h at 35, 39, or 41°C compared with that at 37°C, the standard cultivation temperature.

Similar patterns of affected GO terms related to organelle fission, cell cycle or chromosome organisation and the KEGG pathway DNA replication were found at the three experimental temperatures compared with cultivation at the control temperature. Cultivation at 35°C stimulated transcriptional responses in immune-related pathways, such as cytokine–cytokine receptor interactions and the IL-17 and TNF signaling pathways. Furthermore, cultivation at 35°C leads to an increase in the expression of *RBM3*, which encodes a cold-inducible mRNA binding protein, but not a HSP-related response. At 39°C, in addition to cell growth, other GO terms related to protein-DNA complex subunit organization, ribonucleoprotein binding, vesicle, and Golgi apparatus were found to be enriched, suggesting that myoblasts were more developed and more highly structured at this temperature. Only cultivation at 41°C resulted in increased expression of HSPs, indicative of induced heat shock and DNA damage processing responses. The GO terms and pathways associated with pyrimidine metabolism, cell cycle, DNA replication, and cytoskeleton represent the termination of the proliferative ability and cytoskeletal reorganization in porcine myoblast after 72 h of continuous cultivation at 41 °C. When comparing cells from animals of different ages (P5 vs. P20), most molecular changes were found at the control temperature (37°C), which is the optimal physiological temperature. Although only subtle changes in transcript levels were recorded between P5 and P20 at temperatures both above and below 37°C, we nevertheless identified changes in gene expression patterns that reflect the developmental fate of the myoblasts and influence their responsiveness to thermal stress.

## Data Availability

The datasets presented in this study can be found in online repositories. The expression data are available in the Gene Expression Omnibus public repository with the GEO accession number (GSE202678: GSM6128337- GSM6128360).
